# Assessing negative core beliefs in eating disorders: revision of the Eating Disorder Core Beliefs Questionnaire

**DOI:** 10.1186/s40337-022-00542-9

**Published:** 2022-02-10

**Authors:** Amaani H. Hatoum, Amy L. Burton, Maree J. Abbott

**Affiliations:** 1 School of Psychology, The University of Sydney, Camperdown, NSW Australia; 2 Graduate School of Health, University of Technology Sydney, Ultimo, NSW Australia

**Keywords:** Eating disorders, Core beliefs, Questionnaire, Validity, Factor analysis

## Abstract

**Background:**

Increased theoretical and empirical attention has been given to examining the role of core beliefs in both the development and maintenance of eating disorders (EDs). The Eating Disorder Core Beliefs Questionnaire (ED-CBQ) is self-report measure designed to assess five dimensions of core beliefs relating to eating disorders; self-loathing, unassertive/inhibited, demanding/needing help and support, abandoned/deprived, and high standards for the self. The present study aimed to evaluate the psychometric properties of the ED-CBQ and to develop a revised and improved version of the original measure after evaluating its factor structure and related properties.

**Methods:**

A sample of undergraduate university students (*N* = 763) completed an online test battery of questionnaires. Putative ED-symptomatic (*n* = 384) and non-ED (*n* = 379) subgroups were created from self-reported responses from the Eating Disorder Examination Questionnaire (EDEQ). Confirmatory factor analyses (CFAs) were performed, and internal consistency, construct validity, group differences and clinical utility was examined.

**Results:**

An initial CFA did not support the original five-factor 40-item ED-CBQ. A revised version was developed that possessed equal or superior psychometric properties to the original 40-item measure. The ED-CBQ-R demonstrated superior model fit, similar levels of reliability and construct validity, and the ability to discriminate between putative ED diagnostic groups.

**Conclusions:**

Our results suggest that the ED-CBQ-R is a valid, reliable, but more importantly an efficient and accessible measure with the potential to be utilised both clinically and in research settings.

**Supplementary Information:**

The online version contains supplementary material available at 10.1186/s40337-022-00542-9.

## Plain English Summary

In this study, we evaluated and revised the Eating Disorder Core Beliefs Questionnaire (ED-CBQ); a measure designed to assess five areas of core beliefs hypothesised to be relevant to the development and maintenance of a variety of eating disorder presentations. The present study aimed to evaluate the ED-CBQ and to develop a revised and improved version of the original measure after evaluating its factor structure and related properties. We did not find support for the original ED-CBQ, and as such, a revised version was developed that possessed equal or superior properties to the original 40-item measure. The ED-CBQ-R was able to differentiate between eating disorder symptomatic and non-eating disorder groups, and importantly, was a valid, reliable and efficient tool for future clinicians and researchers. This new tool may prove useful in future research when considering the core cognitive features underlying a variety of disordered eating and feeding presentations.

**Supplementary Information:**

The online version contains supplementary material available at 10.1186/s40337-022-00542-9.

## Introduction

In recent decades, the impact and prevalence of eating disorders (EDs) has increased significantly worldwide [15, 23, 33]. EDs have been associated with increased risk for physical health conditions, including obesity as well as increased psychological distress, role impairment, and have a greater negative impact on interpersonal relationships [26, 29, 34, 38]. Therefore, as a public health concern, it is essential to treat and manage both clinical and subclinical disordered eating behaviours. This is critical particularly in young adult populations shown to be at risk for onset and where prevalence is high [26, 37, 47]. Established treatments would benefit from an increased understanding of the varied factors contributing to the aetiology and maintenance EDs [50].

Increased theoretical and empirical attention has been focused on examining the role of negative core beliefs in both the development and maintenance of EDs [17, 20, 28, 50]. Core beliefs are conceptualised as persistent, pervasive and deeply held beliefs about the self, others or the world [6, 57]. These unconditional and often negative underlying beliefs about the self, others or the world (also known as ‘early maladaptive schemas’) usually develop in childhood or adolescence or as a result of significant life events [6, 57]. Core beliefs have been identified as playing a key role in maintaining cognitive, affective and behavioural symptoms of EDs [6, 49, 57]. Indeed, Fairburn et al.’s [20] transdiagnostic model hypothesised that core low self-esteem is a key factor maintaining over-evaluation of eating, shape and weight and over-evaluation of achieving perfectionism across EDs. Waller’s [50] schema-focussed cognitive-behavioural therapy model of eating disorders predicts that the avoidance of negative affect generated by core beliefs (via the process of schema compensation) facilitates the development of restrictive pathology such that core beliefs triggering affect avoidance contribute to the development of bulimic pathology [50]. Similarly, global negative self-beliefs have been proposed to be the main predisposing factor in the cognitive model of bulimia nervosa (BN) [17], and core low self-esteem the primary underlying predisposing factor for binge eating [11].

Empirical evidence has supported the idea that maladaptive core beliefs relating to EDs are a transdiagnostic factor contributing to increased vulnerability for developing eating psychopathology [48], particularly binge eating, purging, and restriction [28, 36]. Negative self-beliefs have been shown to predict binge eating in both clinical [7] and community samples [56]. More specifically, both binge eating and purging behaviours have been associated with dependence, defectiveness, self-loathing, abandonment, emotional inhibition and deprivation, failure to achieve, insufficient self-control and social isolation core beliefs [1, 28, 35, 36]. Similarly, restrictive pathology has been linked to unrelenting standards, failure to achieve, and social undesirability schemas [35], as well as dependence/incompetence, emotional inhibition and abandonment core beliefs [28, 36].

Clearly it is fundamental that treatments also target the schematic level of processing [50]. Targeting core beliefs in specific ED focused treatment should be assessed for effectiveness in both research and clinical settings [9]. It is therefore essential to have measures of core ED beliefs that are valid, reliable, and accessible, for improving research quality and clinical utility.

A limited number of questionnaires have been developed to adequately measure these beliefs. Perhaps the most widely known is the Young Schema Questionnaire (YSQ; [55], and subsequent short-form versions, which have been extensively used to examine the presence of early maladaptive schemas (e.g., abandonment, emotional inhibition and deprivation). Each of the 16 YSQ subscales has demonstrated validity, reliability and clinical utility with the ability to differentiate between individuals with binge eating disorder (BED) and BN from non-clinical individuals [51, 52].

The Eating Disorder Beliefs Questionnaire (EDBQ; [16] was created to specifically assess three dimensions of assumptions (conditional beliefs) and one of core beliefs (unconditional beliefs) related to eating disorders. The latter dimension—that is, the negative self-beliefs subscale—contains items intended to assess negative core beliefs relating to feelings of inadequacy and defectiveness (e.g., ‘I’m stupid’, ‘I’m a failure’, ‘I’m unlovable’). The EDBQ factor structure has been replicated and it has demonstrated good psychometric properties and ability to distinguish between ED and control participants in several populations [8, 41, 59].

Both the YSQ and EDBQ provide helpful measures of some core beliefs known to be relevant to EDs. However, the YSQ was not developed to identify core beliefs that specifically relate to ED presentations [21], and consequently it does not capture certain relevant core belief dimensions. The most prominent of these is the concept of self-loathing and self-disgust, which has been identified as a theoretically important construct in binge eating [11]. Similarly, the EDBQ is limited in its examination of only one general negative self-beliefs dimension. Hence it does not consider the full range of themes that have been identified to be relevant to individuals with disordered eating [21].

The Eating Disorder Core Beliefs Questionnaire (ED-CBQ; [21] was developed in response to previous measures failing to assess several important core belief constructs. Using both previous theoretical and empirical literature [45, 53], the authors identified several additional and ED specific themes that were not adequately captured by the EDBQ, including defectiveness, rejection, isolation, abandonment, setting of extremely high standards, lack of self-control, and weakness [21]. The ED-CBQ authors also considered that the concepts of self-loathing and self-disgust were particularly important and warranted inclusion in assessing eating disorder relevant core beliefs. Initial items then were selected that were considered characteristic of EDs based on a combination of theoretical judgement, clinical experience and qualitative data [21].

The final 40-item ED-CBQ was developed using an exploratory factor analysis using items across these themes. It assesses five dimensions of core beliefs: (1) Self-loathing (e.g., ‘putrid’), (2) Unassertive/inhibited (e.g., ‘submissive’), (3) Demanding and needing help and support (e.g., ‘needy’), (4) Abandoned/deprived (e.g., ‘abandoned’) and (5) High Standards for Self (e.g., ‘focused’). Subscales exhibited adequate internal consistency (α’s ranged from 0.76 to 0.96). Apart from High Standards for Self, all other subscales demonstrated good convergent validity, as indicated by significant correlations with the Eating Attitudes Test 26 (EAT-26; *r* = 0.26 to 0.38, all *p* < 0.001). The ED-CBQ displayed adequate divergent validity, as indicated by the lack of significant correlation with body mass index (BMI), although it was significantly positively correlated with age. Self-loathing displayed the strongest association with ED diagnostic groups (anorexia nervosa [AN] and BN) relative to the other four subscales.

However, despite these promising initial outcomes, to the best of our knowledge the ED-CBQ has yet to be validated in any population since its preliminary development, and it has only been used in one empirical study (Young and Cooper 2013). Given the renewed interest in the role of underlying core beliefs in the development and maintenance of EDs [3, 38, 44], and the limited information concerning its factor structure, validity, reliability, and clinical utility of this questionnaire, it is timely that an extensive independent assessment of the ED-CBQ be conducted. It is also theoretically important to validate a measure that examines the broadest range of ED core belief dimensions, in order to comprehensively identify the most relevant cognitive constructs associated with eating disorder psychopathology.

### Current study

As such, the aim of the present study was to validate the factor structure and assess the psychometric properties of the ED-CBQ. This included performing a CFA, examining internal consistency, convergent and divergent validity, and examining the ED-CBQ’s ability to distinguish between putative ED-symptomatic and non-ED subgroups, and thereby also testing group differences in negative core beliefs. We also aimed to assess the clinical utility of the ED-CBQ using receiver operating characteristic (ROC) curves, in order to determine optimal clinical cut-off scores. Secondarily, we aimed to create a revised version of the ED-CBQ which had equal or superior psychometric properties to the original 40-item measure, by retaining the best items based off the outcomes of our psychometric evaluation. It is not only essential to have measures of ED core beliefs that are valid and reliable, but importantly *efficient* and accessible, for both improving research quality and increasing clinical utility.

We hypothesised that a CFA would support the original five-factor structure as proposed by Fairchild and Cooper [21], and that the ED-CBQ would display similar psychometric properties and patterns to that of outcomes reported during its original development, including good internal consistency and construct validity. We predicted that we would observe significant positive correlations with related measures of ED symptoms, ED related cognitions and psychological distress and, consistent with prior research, we predicted that there would be no correlations observed between the ED-CBQ and the variables of age and BMI within our sample as eating disorders vulnerability is known to occur across the BMI categories and within a youth sample all individuals would be equally as vulnerable to EDs [21, 29]. However, as seen in the development paper [21], we anticipated High Standards for Self would correlate negatively with the four other subscales and all other included measures. Finally, it was predicted that scores on the ED-CBQ would differ significantly between ED-symptomatic and non-ED subgroups, such that individuals in the ED-symptomatic subgroup would report greater endorsement of all five dimensions of core beliefs.

## Method

### Participants and procedure

All procedures involving human participants were performed in accordance with the ethical standards of The University of Sydney Human Research Ethics Committee (Project Code: 2014_082). The sample of undergraduate university students used in the present study came from a previously published dataset [9]. Although there was no targeted recruitment of high-risk individuals, prevalence of EDs and prodromal disordered eating behaviour has been seen to be high in both men and women in this demographic [26, 37, 47]. All participants were students enrolled in the undergraduate psychology program at The University of Sydney who were invited to participate in the study as part of their studies. After informed consent was obtained, participants voluntarily completed an online test battery of questionnaires using Qualtrics Survey Software in exchange for course credit. This test battery included the measures described in the materials section and additional demographic information presented in a randomised order. Data from 763 participants was included in the present study (71% female, *M*_*age*_ = 19.36, *SD* = 3.47), in order to validate the factor structure and psychometric properties of the ED-CBQ and examine its ability to discriminate between ED-symptomatic and non-ED subgroups.

A putative ED-symptomatic subgroup of participants (*n* = 384 [50.3%]; 82.6% female; *M*_*age*_ = 19.21, *SD* = 3.21) was created based off their self-reported responses from the Eating Disorder Examination Questionnaire (EDE-Q). We intended to create a group representing individuals with both clinical and sub-clinical (or prodromal) symptomatology, and other specified feeding and eating disorders (OSFED), and as such considered the frequently used clinical cut-off score of 4 to be too exclusionary [19]. More recent literature has suggested that optimal EDE-Q clinical cut-off scores vary from 1.68 to 2.93 [32, 39, 40, 42]. To arrive at a cut-off score that would best capture eating disorder symptomatology, including OSFED, we considered the score that would most closely represent the DSM-5 criterion for FED diagnoses (5th ed.; DSM-5; [2]). Thus, a global EDE-Q score of 2.5 or higher was used as a cut-off criterion for the ED-symptomatic.

There was no significant difference in age between the ED-symptomatic and non-ED subgroups, *F*(1, 761) = 1.464, *p* = 0.227. However, the proportion of females compared to males was significantly higher in the ED-symptomatic subgroup (82.6% female) compared to the non-ED subgroup (59.4% female), *X*^2^ (2, *N* = 571) = 50.790, *p* < 0.001.

### Materials

#### Eating Disorder Core Beliefs Questionnaire (ED-CBQ)

The ED-CBQ is a 40-item self-report measure that assesses core beliefs relating to EDs [21]. It contains five subscales: Self-loathing (10 items), Unassertive/inhibited (eight items), Demanding/needing help and support (10 items), Abandoned/isolated (four items), and High Standards for Self (eight items). Items were rated on a 7-point scale from *Feels very much untrue* (1) to *Feels very much true* (7), where a higher mean score reflected higher ED core beliefs.

#### Eating Disorder Examination Questionnaire (EDE-Q)

The EDE-Q is a 28-item self-report questionnaire that examines the frequency and severity of ED symptoms experienced 28 days prior to assessment [18]. It contains four subscales: dietary restraint, eating concern, weight concern and shape concern, and items relating to the frequency and severity of binge eating episodes (item 13). Items forming part of one of the four subscales were either rated on a 7-point scale from *No days* (0) to *Every day* (6) or participants recorded the number of times a symptom was experienced. A higher global score (four subscales) reflects greater frequency and severity of symptoms. In the present study, a score of 2.5 or higher was used to create the putative ED-symptomatic. The EDE-Q is considered the gold-standard self-report assessment tool for examining ED symptomatology [24]. The four subscales and the overall EDEQ demonstrated good internal consistencies in the present study (α = 0.80 to 0.97).

#### Eating Disorder Beliefs Questionnaire (EDBQ)

The EDBQ is a 32-item self-report questionnaire that examines core beliefs and underlying assumptions relating to the development and maintenance of EDs [16]. It contains four subscales: negative self-beliefs (10 items: e.g., ‘I’m a failure.’), acceptance by others (10 items: e.g., ‘If I lose weight, people will care about me.’), self-acceptance (six items: ‘If my stomach is flat, I’ll be more desirable.’), and control over eating (six items: e.g., ‘If I eat normally, I’ll gain weight.’). Items are rated on a scale from 0 *(I do not usually believe this at all)* to 100 *(I am usually completely convinced that this is true)*, where higher scores indicate stronger beliefs or assumptions. Previous validations suggest the EDBQ has good psychometric properties [8, 41]. The four subscales and the overall EDBQ demonstrated good internal consistencies in the present study (α = 0.88 to 0.97).

#### Eating Beliefs Questionnaire 18 (EBQ-18)

The EBQ-18 is an 18-item self-report questionnaire that examines both positive (six items: e.g., ‘Eating helps to control my emotions’) and negative beliefs (six items: e.g., ‘Once I start eating, I can’t stop’) and permissive beliefs (six items: e.g., ‘I like to binge’) about food and eating in the absence of hunger [10]. Items are rated on a scale from *Strongly disagree* (1) to *Strongly agree* (5). The EBQ-18 has been found to be a valid, reliable and clinically useful self-report measure [12]. The three subscales and the overall EBQ-18 demonstrated good internal consistencies in our sample (α = 0.87 to 0.92).

#### Depression Anxiety Stress Scales 21 (DASS-21)

The DASS-21 is a 21-item self-report questionnaire that examines three related negative emotional states; depression (e.g., ‘I felt that life was meaningless’), anxiety (e.g., ‘I felt I was close to panic’) and stress (e.g., ‘I found it hard to wind down’; [30]. Participants were asked to rate items according to how much each statement applied to them over the past week on a scale of 0 (*Did not apply to me at all*) to 3 (*Applied to me very much or most of the time*), where higher scores indicate higher depression, anxiety and stress. The scale has demonstrated good psychometric properties across cultures [58]. The three subscales and the overall DASS-21 demonstrated good internal consistencies in our sample (α = 0.80 to 0.93).

### Statistical analyses

Statistical analyses were carried out using IBM Statistical Package for Social Sciences (SPSS) Statistics (version 26.0), predictive analytics software. The overall distribution of data was examined to assess for violations of normality assumptions for all variables for the subgroups and the sample as a whole. There were no missing values in the dataset used in the present study.

CFA’s were conducted in IBM SPSS Amos (version 26.0; [4] to validate the five-factor structure of the original ED-CBQ model and the subsequent revised models, in both the full sample and with the ED-symptomatic subgroup. We allowed all latent variables to covary, maximum likelihood estimation was used with an unbiased covariance matrix as input, and there were no missing values. Errors were not correlated, and no modifications were made based off modification indices. Model fit was evaluated based off the following cut-off values as suggested in the literature [25, 27, 31, 43],a comparative fit index (CFI of ⩾ 0.95 was considered good and ⩾ 0.90 acceptable, a χ^2^/df value of ⩽ 2.00 considered good and ⩽ 3.00 acceptable, and an RMSEA value of ⩽ 0.050 considered good and ⩽ 0.080 acceptable.

The internal consistency of the subscales and overall scales were assessed using Cronbach’s α, with values > 0.70 considered to be acceptable [46]. Pearson’s correlations were used to examine intercorrelations between subscales and convergent and divergent validity. The DASS-21, EDE-Q, EDBQ and EBQ-18 were used to assess convergent validity, and age and body mass index (BMI) to test for divergent validity. As the EDE-Q global and subscale scores do not encapsulate binge eating which we determined is an important feature of the presentation of eating disorders to consider in evaluating the ED-CBQ, we also observed the correlations between the item 13 from the EDE-Q (which assesses the frequency of binge eating) and the ED-CBQ. Differences between the ED-symptomatic and non-ED groups were examined using independent sample *t*-tests and one-way analysis of variance (ANOVA).

ROC curve analyses were conducted using the MedCalc program (MedCalc Software, Mariakerke, Belgium) to determine optimal clinical cut-off scores and other indicators of test performance. These included determining area under the curve (AUC), sensitivity (indication of the likelihood of true positives), specificity (indication of the likelihood of false negatives) as well as positive predictive value (PPV; probability the disorder is present when the test is positive) and negative predictive value (NPV; probability the disorder is not present when the test is negative). An AUC value closer to 1.0 than to 0.5 indicates that the model more reliably separates the two groups; the higher the AUC value the more likely that the criterion value will be able to differentiate the two groups.

## Results

### Confirmatory factor analyses

An initial CFA was conducted to validate the five-factor structure of the ED-CBQ. Using the previously indicated cut-off values (see “Method”), it was evident the original five-factor model did not provide an acceptable fit to the observed data for either the full sample or the ED-symptomatic subgroup (see Table 1). In the full sample, items loaded adequately (β > 0.30; [13] onto their intended factor, except the item ‘*Painstaking*’ (β = − 0.03). In the ED-symptomatic subgroup, items loaded adequately, except for the items ‘*Painstaking*’ (β = − 0.07) and ‘*Meticulous’* (β = 0.27). See Additional file 2: Table A for all factor loadings, communalities and Cronbach’s α’s if items were removed.

**Table 1:** Model fit indices for the original five-factor model of the ED-CBQ and the Four-factor model of the ED-CBQ-R

Fit index	ED-CBQ Full sample (*n* = 763)	ED-CBQ ED-symptomatic subgroup (*n* = 384)	ED-CBQ-R Full sample (*n* = 763)	ED-CBQ-R ED-symptomatic subgroup (*n* = 384)
χ^2^/df	5.30	3.31	3.19	1.98
CFI	.79	.77	.97	.97
TLI	.77	.76	.96	.96
NFI	.75	.70	.95	.94
RMSEA	.075	.078	.054	.051
[90% CI]	[.073, .077]	[.074, .081]	[.047, .061]	[.039, .062]

Absolute fit indices: χ^2^/df = chi-square/degrees of freedom. ED = Eating Disorder; ED-CBQ = Eating Disorder Core Beliefs Questionnaire; ED-CBQ-R = Eating Disorder Core Beliefs Questionnaire Revised; Comparative fit indices: CFI = Comparative fit index; TLI = Tucker-Lewis index; NFI = normed fit index. RMSEA = root mean square error of approximation; CI = confidence interval

In response to these outcomes and in accordance with our secondary aim, we developed a revised version of the ED-CBQ (ED-CBQ-R). We aimed to develop a revised model with superior goodness of fit, reliability and validity, and a measure with increased efficiency and clinical utility. Several subsequent models were tested in this process.^1^ A final four-factor 15-item model was created by examining the model fit indices, standardised regression coefficients (removing items incrementally with a β < 0.50), item communalities, internal consistencies of each subscale (including Cronbach’s α’s if items were removed) and by using theoretical guidance and judgement. At least three items per subscale were retained to create the resultant model in alignment with best practice guidelines [13].

Using the established cut-off values, the resultant four-factor structure of the ED-CBQ-R demonstrated an acceptable to good fit to the observed data in both the full sample and the ED-symptomatic subgroup (See Table 1). All items displayed high loadings onto their intended factor (all above 0.55) and communalities (all > 0.20; [14]. See Additional file 1: File 1 for details of the final measure, and Additional file 3: Table B all factor loadings, communalities and Cronbach’s α’s if items were removed.

### Internal consistency

#### ED-CBQ

Across the full sample and both the ED-symptomatic and non-ED subgroups, the total ED-CBQ and Self-loathing had good internal consistency (see Table 2). Using the full sample (*N* = 763), Cronbach’s α was predicted to increase if the items *Focused, Goal-oriented,* and *Self-disciplined* were removed from the overall scale, as it did for the item *Goal-oriented* in the ED-symptomatic subgroup (refer to Additional file 2: Table A for more detail). Demanding displayed good internal consistency across subgroups, and Unassertive acceptable internal consistency, with Cronbach’s α predicted to increase if the item *Unemotional* was removed. Abandoned demonstrated acceptable internal consistency, with Cronbach’s α predicted to increase if the item *Misunderstood* was removed. High Standards for Self also had acceptable internal consistency, with Cronbach’s α predicted to increase if the item *Painstaking* was removed. See Table 2 for Cronbach’s α’s.

**Table 2:** Internal consistencies of the ED-CBQ and ED-CBQ-R in the full sample, ED-symptomatic and non-ED subgroups

	Full sample (*N* = 763)	ED-symptomatic subgroup (*n* = 384)	Non-ED subgroup (*n* = 379)
**Total ED-CBQ**	α = .92	α = .92	α = .90
Self-loathing	α = .93	α = .93	α = .90
Unassertive	α = .80	α = .78	α = .81
Demanding	α = .85	α = .85	α = .82
Abandoned	α = .77	α = .76	α = .71
High standards for self	α = .76	α = .75	α = .78
**Total ED-CBQ-R**	α = .89	α = .88	α = .86
Self-loathing	α = .92	α = .93	α = .89
Unassertive	α = .74	α = .71	α = .76
Demanding	α = .75	α = .75	α = .72
Abandoned	α = .78	α = .78	α = .71

ED = Eating Disorder; ED-CBQ = Eating Disorder Core Beliefs Questionnaire; ED-CBQ-R = Eating Disorder Core Beliefs Questionnaire Revised

#### ED-CBQ-R

In the full sample and both subgroups, the total ED-CBQ-R and Self-loathing displayed good internal consistency. All other subscales displayed adequate internal consistency (see Table 2). In the full sample and in both subgroups, Cronbach’s α was predicted to increase if the item *Deprived* was removed from the Abandoned subscale (refer to Additional file 2: Table A for more detail). Due to the need to retain at least three items per subscale, this item was retained in the revised measure.

### Subscale inter-correlations

#### ED-CBQ

All ED-CBQ subscales were significantly positively correlated with the overall scale (*r* = 0.35** to 0.86**). Four of five subscales were significantly positively correlated with each other (*r* = 0.50** to 0.61**). Only High Standards for Self displayed a different pattern of relationships with the other subscales. It did not significantly correlate with Self-loathing (*r* = -0.07) and demonstrated weak positive correlations with all other subscales (*r* = 0.09* to 0.18**).

#### ED-CBQ-R

The total ED-CBQ-R was significantly positively correlated with all four of its subscales (*r* = 0.73** to 0.82**), and all four subscales positively correlated with each other (*r* = 0.40** to 0.55**).

### Convergent validity

#### ED-CBQ

The total ED-CBQ, Self-loathing, Demanding, Unassertive and Abandoned subscales were all significantly positively correlated with all other included measures (DASS-21, EBQ, EDBQ, EDE-Q) and their respective subscales. High Standards for Self was not significantly correlated with the majority of other measures included. This subscale only displayed weak positive correlations with the EDBQ self-acceptance subscale and DASS-21 stress subscale, and weak negative correlations with the DASS-21 depression subscale and the EBQ-18 permissive beliefs subscale. See Table 3 for all correlations.

**Table 3:** Correlations of the Total ED-CBQ and ED-CBQ-R and Subscales with Other Included Measures in the Full Sample (N = 763)

	Total ED-CBQ	Self-loathing	Unassertive	Demanding	Abandoned	High standards for self	Total ED-CBQ-R	Self-loathing	Unassertive	Demanding	Abandoned
Age	.02	.01	.01	-.02	.08*	.05	.03	.02	.04	-.05	.10**
BMI	.03	.14**	-.04	.021	.03	-.08*	.04	.14**	-.07*	.04	.03
DASS-21-depression	.54**	.52**	.42**	.48**	.59**	-.10**	.60**	.43**	.43**	.47**	.55**
DASS-21-anxiety	.52**	.47**	.39**	.50**	.49**	.03	.56**	.43**	.41**	.44**	.47**
DASS-21-stress	.55**	.45**	.34**	54**	.53**	.09*	.58**	.40**	.38**	.53**	.50**
**EBQ-18 Total**	.42**	.41**	.33**	.41**	.37**	-.07	.47**	.39**	.33**	.37**	.37**
EBQ-18-Positive	.38**	.33**	.29**	.36**	.37**	-.00	.43**	.32**	.30**	.34**	.37**
EBQ-18-Negative	.42**	.43**	.29**	.41**	.38**	-.07	.46**	.41**	.29**	.38**	.38**
EBQ-18-Permissive	.21**	.23**	.21**	.22**	.12**	-.09**	.23**	.21**	.21**	.17**	.13**
**EDBQ Total**	.59**	.59**	.41**	.51**	.58**	-.03	.64**	.55**	.41**	.49**	.56**
EDBQ-Negative	.62**	.60**	.50**	.52**	.66**	-.06	.69**	.53**	.50**	.52**	.62**
EDBQ-Self-acceptance	.41**	.34**	.25**	.38**	.38**	.08*	.42**	.31**	.25**	.39**	.37**
EDBQ-Other-acceptance	.46**	.51**	.30**	.41**	.43**	-.04	.50**	.48**	.29**	.38**	.43**
EDBQ-Eating Control	.46**	.52**	.32**	.38**	.43**	-.05	.51**	.51**	.31**	.34**	.45**
**EDE-Q Global**	.39**	.42**	.23**	.34**	.41**	-.01	.43**	.40**	.23**	.33**	.41**
EDE-Q-Restraint	.24**	.28**	.12**	.19**	.25**	.03	.25**	.27**	.11**	.17**	.25**
EDE-Q-Eating Concern	.39**	.43**	.24**	.33**	.42**	-.03	.44**	.43**	.24**	.31**	.43**
EDE-Q-Weight Concern	.35**	.39**	.20**	.30**	.36**	-.01	.40**	.38**	.20**	.30**	.37**
EDE-Q-Shape Concern	.39**	.39**	.25**	.35**	.42**	-.01	.44**	.37**	.25**	.35**	.42**

BMI = Body Mass Index; DASS-21 = Depression Anxiety Stress Scale 21; EBQ-18 = Eating Beliefs Questionnaire 18; EDBQ = Eating Disorder Beliefs Questionnaire; ED = Eating Disorder; ED-CBQ = Eating Disorder Core Beliefs Questionnaire; ED-CBQ-R = Eating Disorder Core Beliefs Questionnaire Revised; EDE-Q = Eating Disorder Examination Questionnaire; SD = Standard Deviation

*p < .05 (two-tailed). **p < .01 (two-tailed)

We also examined the relationship between the ED-CBQ and its subscales with binge frequency, as determined by self-reported scores on the EDE-Q. The total ED-CBQ (*r* = 0.17**), Self-loathing (*r* = 0.20**), Demanding (*r* = 0.20**), and Abandoned (*r* = 0.14**) were all significantly positively correlated with binge frequency, whereas Unassertive (*r* = 0.04) and High Standards for Self (*r* = -0.02) were not significantly correlated with binge frequency.

#### ED-CBQ-R

The total ED-CBQ and the total ED-CBQ-R were significantly positively correlated (*r* = 0.93**). The total ED-CBQ-R, Self-loathing, Demanding, Unassertive and Abandoned were all significantly positively correlated with all other included measures and their respective subscales (see Table 3). Additionally, the total ED-CBQ-R (*r* = 0.17**), Self-loathing (*r* = 0.18**), Demanding (*r* = 0.17**), and Abandoned (*r* = 0.15**) were all significantly positively correlated with binge frequency. Only Unassertive was not significantly correlated with binge frequency (*r* = 0.03).

### Divergent validity

#### ED-CBQ

Consistent with the original ED-CBQ development paper [21], we examined the correlations between age and body mass index (BMI) and the total ED-CBQ and its subscales to test for divergent validity in our sample as these variables were not expected to correlate with the presence or severity of eating disorder cognitions within our sample [29]. The total ED-CBQ, Self-loathing, Unassertive, Demanding and High Standards for Self were not significantly correlated with age. Only the abandoned subscale displayed a weak significant positive correlation with age. The total ED-CBQ, Unassertive, Demanding and Abandoned subscales were not significantly correlated with BMI. However, Self-loathing was significantly positively correlated with BMI, and High Standards for Self displayed a weak negative correlation with BMI.

#### ED-CBQ-R

The total ED-CBQ-R was not significantly correlated with age or BMI. Self-loathing, Unassertive, and Demanding were not significantly correlated with age, although Abandoned displayed a weak positive correlation with age. Demanding and Abandoned were not significantly correlated with BMI. However, Self-loathing and Abandoned were significantly positively correlated with BMI, and Unassertive displayed a weak negative correlation with BMI. See Table 3 for correlations.

### Descriptive statistics

#### ED-CBQ

Of the possible range of scores (1 to 7), scores on the overall ED-CBQ in our sample ranged from 1 (0.4%) to 6.68 (0.1%), from 1 (27.1%) to 7 (0.1%) on Self-loathing, from 1 (3.9%) to 6.5 (0.1%) on Unassertive, from 1 (10.2%) to 7 (0.1%) on Abandoned, from 1 (1.8%) to 6.6 (0.1%) on Demanding, and from 1 (0.7%) to 7 (0.1%) on High Standards for Self.

#### ED-CBQ-R

Scores on the total ED-CBQ-R in our sample ranged from 1 (2%) to 6.67 (0.1%), from 1 (54.3%) to 7 (0.4%) on Self-loathing, from 1 (10.4%) to 7 (0.1%) on Unassertive, from 1 (27.7%) to 7 (0.5%) on Abandoned, and from 1 (5.1%) to 6.6 (0.1%) on Demanding. See Table 4 for all relevant descriptive statistics.

**Table 4:** Descriptive Statistics for the ED-CBQ and ED-CBQ-R in the Full Sample, ED-symptomatic and non-ED Subgroups

	Full sample (*N* = 763)	ED-symptomatic subgroup (*n* = 384)	Non-ED subgroup (*n* = 379)	*t*	*p*	*d*
**Total ED-CBQ Mean (SD)**	3.04 (0.82)	3.29 (0.84)	2.78 (0.71)	9.00	< .001	.65
Self-Loathing Mean (SD)	1.85 (1.08)	2.17 (1.25)	1.52 (0.76)	8.59	< .001	.62
Unassertive Mean (SD)	3.04 (1.13)	3.26 (1.08)	2.82 (1.14)	5.46	< .001	.40
Demanding Mean (SD)	3.23 (1.19)	3.54 (1.22)	2.92 (1.07)	7.57	< .001	.55
Abandoned Mean (SD)	2.82 (1.37)	3.29 (1.42)	2.34 (1.13)	10.18	< .001	.74
High Standards for Self Mean (SD)	4.39 (1.05)	4.40 (1.05)	4.39 (1.06)	0.29	.775	.02
**Total ED-CBQ-R Mean (SD)**	2.71 (1.05)	3.06 (1.08)	2.35 (0.89)	9.94	< .001	.72
Self-Loathing Mean (SD)	1.75 (1.19)	2.07 (1.40)	1.42 (0.81)	7.82	< .001	.57
Unassertive Mean (SD)	3.12 (1.37)	3.38 (1.30)	2.86 (1.38)	5.37	< .001	.39
Demanding Mean (SD)	3.45 (1.40)	3.82 (1.42)	3.07 (1.28)	7.66	< .001	.56
Abandoned Mean (SD)	2.45 (1.46)	2.94 (1.56)	1.95 (1.16)	9.99	< .001	.72

*t*-values indicates the difference in scores between the ED-symptomatic and non-ED subgroups. ED = Eating Disorder; ED-CBQ = Eating Disorder Core Beliefs Questionnaire; ED-CBQ-R = Eating Disorder Core Beliefs Questionnaire Revised; SD = Standard Deviation. Scores ranged from 1 to 7, with higher scores indicating higher endorsement of each subscale

### Group differences

#### ED-CBQ

Results from independent samples *t*-tests revealed that participants in the ED-symptomatic subgroup scored significantly higher on the original 40-item ED-CBQ total scale than those in the non-ED subgroup (see Table 4 for all relevant inferential statistics). Participants in the ED-symptomatic subgroup also scored significantly higher than the non-ED subgroup on Self-loathing, Unassertive, Abandoned, and Demanding subscales. However, there was no significant difference between the ED-symptomatic and non-ED subgroups on High Standards for Self.

#### ED-CBQ-R

Participants in the ED-symptomatic subgroup scored significantly higher than those in the non-ED subgroup on the total ED-CBQ-R, and Self-loathing, Unassertive, Abandoned, and Demanding subscales (see Table 4).^2^

### ROC curves

#### ED-CBQ

The cut-off scores for the total ED-CBQ, Self-loathing, Demanding, Unassertive and Abandoned all had significant AUC values, indicating these cut-off values function better than chance at discriminating between individuals with clinical or sub-clinical eating disorder symptomatology compared to those without. The AUC value for High Standards for Self was not significant, which indicated that this subscale may not be a reliable indicator of differences between these groups of individuals. The cut-off criterions reported was one for which both the sensitivity and specificity was maximal (using the Youden-Index; [22]. Table 5 outlines the Youden Index for each subscale, as well as all other included test performance indicators using the comparison sample of the ED-symptomatic (*n* = 384) versus non-ED (*n* = 379) subgroups.

**Table 5:** Results from ROC Curve Analyses for the ED-CBQ and ED-CBQ-R

Scale	Youden Index	Sensitivity (95% CI)	Specificity (95% CI)	PPV (95% CI)	NPV (95% CI)	AUC (95% CI)
**ED-CBQ Total**	> 2.88	69.01 (64.1–73.6)	59.37 (54.2–64.4)	63.20 (60.0–66.4)	65.40 (61.4–69.2)	0.680** (0.645–0.713)
Self-loathing	> 1.70	51.04 (45.9–56.1)	75.73 (71.1–80.0)	68.10 (63.5–72.3)	60.40 (57.6–63.2)	0.674** (0.639–0.707)
Demanding	> 3.80	44.79 (39.7–49.9)	79.95 (75.6–83.9)	69.40 (64.3–74.0)	58.80 (56.3–61.3)	0.651** (0.616–0.685)
Unassertive	> 2.63	69.79 (64.9–74.3)	47.76 (42.6–52.9)	57.50 (54.6–60.3)	60.90 (56.5–65.2)	0.609** (0.573–0.643)
Abandoned	> 2.75	57.29 (52.2–62.3)	73.61 (68.9–78.0)	68.70 (64.6–72.7)	63.00 (59.9–66.0)	0.696** (0.662–0.728)
High Standards for Self	> 3.50	80.99 (76.7–84.8)	23.22 (19.1–27.8)	51.70 (49.8–53.5)	54.70 (47.8–61.4)	0.507 (0.471–0.543)
**ED-CBQ-R Total**	> 2.80	59.11 (54.0–64.1)	71.77 (66.9–76.2)	68.0 (63.9–71.8)	63.4 (60.2–66.5)	0.693** (0.659–0.726)
Self-loathing	> 1.25	51.04 (45.9–56.1)	73.88 (69.1–78.2)	66.40 (61.9–70.7)	59.80 (57.0–62.6)	0.646** (0.611–0.680)
Demanding	> 3.75	51.82 (46.7–56.9)	72.3 (67.5–76.7)	65.50 (61.1–69.6)	59.70 (56.8–62.6)	0.654** (0.619–0.688)
Unassertive	> 2.75	64.32 (59.3–69.1)	52.51 (47.3–57.6)	57.80 (54.7–61.0)	59.10 (55.2–63.1)	0.607** (0.571–0.642)
Abandoned	> 2.33	55.73 (50.6–60.8)	77.57 (73.0–81.7)	71.60 (67.2–75.6)	63.40 (60.4–66.2)	0.689** (0.655–0.711)

Analyses were conducted using the ED-symptomatic (*n* = 384) versus non-ED (*n* = 379) subgroups. Scores ranged from 1 to 7, with higher scores indicating higher endorsement of each subscale. AUC = Area under the curve; CI = Confidence Interval; ED = Eating Disorder; ED-CBQ = Eating Disorder Core Beliefs Questionnaire; ED-CBQ-R = Eating Disorder Core Beliefs Questionnaire Revised; NPV = Negative predictive value; PPV = Positive predictive value; ROC = Receiver operating characteristic curve

**p < .001

#### ED-CBQ-R

Further, the optimal criterion (Youden Index) identified for each the ED-CBQ-R and each of its subscales had significant AUC values, indicating these cut-off values function better than chance at discriminating between individuals with clinical or sub-clinical eating disorder symptomatology compared to those without (see Table 5).

## Discussion

The present study aimed to evaluate the psychometric properties of the ED-CBQ and to develop a revised and improved version of the original measure, after evaluating its factor structure and related properties. Contrary to our primary hypothesis, results did not support the original five-factor 40-item ED-CBQ model as proposed by Fairchild and Cooper [21]. However, the ED-CBQ total scale score displayed acceptable internal consistency and construct validity, and the ED-symptomatic reported significantly greater endorsement of four of five dimensions of core beliefs compared to the non-ED subgroup.

In response to the outcomes of our primary evaluation of the factor structure of the ED-CBQ and to address our secondary aim, a revised version was developed that possesses equal and/or superior psychometric properties to the original 40-item measure. The ED-CBQ-R demonstrates superior model fit compared to the ED-CBQ, similar levels of reliability and construct validity, the ability to discriminate between putative ED-symptomatic groups, and increased practical and theoretical value.

### ED-CBQ

It was evident the five-factor structure of the original 40-item ED-CBQ was not supported by our data. Several items only displayed adequate loadings and two items did not load onto their intended factor [13]. Our results also indicated that although all subscales demonstrated acceptable internal consistency, Self-loathing was the most reliable, as it was in the development paper [21]. Notably, removing several items from High Standards for Self increased the reliability of the overall ED-CBQ. Further, all subscales were strongly positively correlated, apart from High Standards for Self, which did not correlate with Self-loathing and had only weak positive correlations with the other subscales. The overall ED-CBQ and four of five subscales demonstrated good convergent validity and adequate divergent validity. Only High Standards for Self did not demonstrate good construct validity. Furthermore, it is interesting to note that (with the notable exception of the High Standards for Self subscale) the correlations between the ED-CBQ and the other related cognitive measures (the EBQ-18 and the EDBQ) largely ranged between 0.2 and 0.6 with no correlations between these measures greater than 0.7 indicating that these measures are distinct but related measures.

Individuals in the ED-symptomatic subgroup reported significantly higher scores on the total ED-CBQ, Self-loathing, Unassertive, Abandoned and Demanding compared to those in the non-ED subgroup. This supports both findings from the development paper [21] and previous literature which has evidenced the relationship between these core beliefs and binge eating, purging and restrictive behaviours in a variety of clinical ED presentations [28]. Scores on High standards for Self did not significantly differ between the ED-symptomatic and non-ED subgroups. Interestingly, this subscale was endorsed the most highly on average compared to any other subscale, indicating all participants equally endorsed having relatively high standards for themselves. As suggested by existing theoretical and empirical literature, having high standards for the self remains a theoretically important construct for individuals with particular ED presentations [5, 28]. However, the subscale was unable to discriminate between subgroups in our sample. Moreover, it did not correlate significantly with binge frequency. This finding does not support previous literature that has suggested unrelenting standards and perfectionistic tendencies have been associated with binge eating [28].

Taken together, this pattern of results indicates that having high standards for oneself appears to be conceptually distinct to beliefs of self-loathing, feeling abandoned, or believing oneself to be demanding or unassertive in the context of eating disorders. As such, we propose a possible explanation for this overall pattern. Each of the latter concepts represents of a pattern of maladaptive core beliefs characterised by endorsing a negative or undesirable view of the self. In contrast, having high or unrelenting standards for oneself appears comparatively more positive or desirable. As such, this construct may have better aligned with the other more negative dimensions of core beliefs if phrased in a way that individuals perceived themselves to be failing to meet those standards. The literature has indicated that although individuals with EDs may ultimately have higher standards for themselves, those individuals displaying binge eating, purging and restrictive behaviours are more self-critical and as such may perceive themselves to have insufficient self-control or to have failed to achieve those standards [5, 28, 48]. Thus, it is possible that a concept with more negative valence would have not only better captured this dimension of core beliefs in ED-symptomatic individuals, but also increased inter-subscale correlations, and ultimately the internal consistency and validity of the overall scale.

Optimal clinical cut-off scores for the ED-CBQ were also identified in order to provide new information regarding its potential clinical utility. The significant AUC values suggest that even the relatively low Youden index cut-off scores identified for Self-loathing, Demanding, Unassertive and Abandoned can reliably discriminate between individuals with clinically significant feeding and eating disorder symptomatology compared to those without. ROC curve analyses did not support the use of High Standards for Self to differentiate between these groups of individuals. It is important to note however, that these scores cannot be utilised in place of examining DSM-5 diagnostic criteria but can instead provide important supplementary information by identifying clinically significant treatment targets (i.e., core beliefs) for those who have been diagnosed with an eating or feeding disorder.

### ED-CBQ-R

The final four-factor ED-CBQ-R demonstrated superior model fit, higher factor loadings, and better communalities in the full sample and ED-symptomatic subgroup compared to the original ED-CBQ. Overall, the reliability of each revised subscale was comparable to that of the original measure (all α > 0.70). The internal consistency of the overall ED-CBQ-R (α = 0.86 to 0.89) was also comparable to that of the original ED-CBQ, despite reducing the overall number of items. Further, in the revised measure all subscales were positively correlated, whereas in the original measure High Standards for Self was negatively correlated with all four other subscales. This improves the interpretability the total ED-CBQ-R, and results in a measure with greater statistical and theoretical consistency.

The overall scale also displayed excellent construct validity, as evidenced by the consistent significant positive correlations with the wide variety of related measures, and not being significantly correlated with age or BMI. Moreover, the total ED-CBQ-R, Self-loathing, Demanding and Abandoned subscales were all positively correlated with binge frequency. This is consistent with previous empirical research that has found abandonment, dependence, shame and social undesirability schemas were related to binge eating in individuals diagnosed with BED and BN [28]. Unassertive was not significantly correlated with binge frequency, which is an unexpected result given that previous research has found that the related construct of emotional inhibition was associated with clinically significant binge eating [28]. Once more, it is important to note that correlations between the ED-CBQ-R and the other related cognitive measures (the EBQ-18 and the EDBQ) largely ranged between 0.2 and 0.7 with no correlations between these measures greater than 0.7 providing evidence that these measures are distinct but related measures.

Further, the ED-CBQ-R was able to discriminate between ED-symptomatic and non-ED subgroups, and the significant AUC values identified using ROC curve analyses suggested that the Youden index cut-off scores for the ED-CBQ-R and all four subscales can be used to reliably differentiate between ED-symptomatic and non-ED subgroups. In all, the ED-CBQ-R represents a refined, brief, and efficient measure with strong psychometric properties, that remains clinically useful and easily interpretable, but is still able to examine four diverse core belief dimensions pertinent to transdiagnostic eating disorder related symptoms and behaviours.

### Theoretical and clinical implications

The present research has both theoretical and clinical significance. Theoretically, the outcomes of our studies have validated the importance of self-loathing, feeling abandoned or isolated, believing oneself to be demanding, needing help or being unassertive or emotionally inhibited for individuals reporting clinical or sub-clinical eating disorder symptomatology. We have provided evidence supporting the relationship between these specific ED core belief dimensions and the presence of eating, weight and shape concerns and restriction and binge eating. Further, although having high or unrelenting standards for oneself remains a critically important construct in individuals displaying these concerns and behaviours, our research provides theoretical support for the idea that these individuals may also be more self-critical or ultimately perceive themselves to be failing to meet those standards. Thus, to best capture this dimension in individuals with FED, it may be pertinent to investigate either the aspiration towards those standards or alternatively the perceived failure to meet them.

Practically, previously only the YSQ and EDBQ have been utilised extensively in research to identify maladaptive ED core beliefs. This research provides the first psychometric evaluation of the ED-CBQ, as the only existing measure designed to solely and specifically assess ED core beliefs. This evaluation ultimately allowed us to develop a revised scale with increased practical and clinical utility, which possesses psychometric properties equal or superior to that of the original scale. The ED-CBQ-R provides both theoretical and practical advantages, by possessing superior factor structure, equal internal consistency and construct validity, and importantly greater interpretability overall compared to the ED-CBQ. It allows researchers and clinicians to comprehensively assess the most relevant cognitive constructs associated with eating disorder psychopathology, whilst still being a brief, valid and reliable measure. Given maladaptive core beliefs may be key transdiagnostic factors increasing vulnerability to the development and maintenance of EDs [48], in conjunction with standard assessment of ED symptomatology, this new measure may be a promising tool for identifying and monitoring treatment targets in both cognitive behavioural and schema therapy.

### Limitations and future directions

Notwithstanding the theoretical and practical significance of this research, there are several limitations worth noting. Firstly, our study was conducted using a non-clinical sample of university students. As such, the ED-symptomatic subgroup was not created on the basis of a formal diagnosis or clinical interview but on self-reported outcomes of participants. Further, as a core beliefs measure, we would theoretically expect a high level of stability of self-reported outcomes on all ED-CBQ dimensions [54]. However, we were unable to assess test re-test reliability. To address these limitations, future research should replicate the current research in a clinical group with a focus on assessing temporal stability of outcomes.

It would be also interesting for future research to address other gaps and questions left unanswered by the present research, such as examining potential differences between specific ED subgroups (i.e., differentiating subscales between AN, BN, BED and OSFED groups) and possible gender differences in outcomes. We did not examine gender differences, as like much of ED research, the vast majority of our sample was female. Future research should certainly emphasise the recruitment of male participants for more focussed investigation. Finally, although this was only a preliminary validation of the EDCBQ-R, promising outcomes from the present study indicate this research warrants future replication in treatment-seeking sample. It would also be pertinent to validate the ED-CBQ-R across diverse age-groups in order to compare the presence of different core belief constructs across developmental stages to better understand their role as predisposing and maintaining factors.

## Conclusions

In all, we extend existing literature by providing the first evaluation of the factor structure and psychometric properties of the ED-CBQ since its development. We also present a revised version of the original measure. The current findings suggest that the ED-CBQ-R is a valid, reliable, and importantly an efficient and accessible measure with the potential to be utilised both clinically and in research settings. It is critical to continue to utilise validated cognitive assessment tools alongside diagnostic and symptomatic measures. The ED-CBQ-R may also prove useful in future theoretical and empirical research when considering the core cognitive features underlying a variety of disordered eating and feeding presentations.

## Supplementary Information

**Additional file 1: File 1.** Eating Disorder Core Beliefs Questionnaire - Revised (ED-CBQ-R).

**Additional file 2: Table A.** Confirmatory Factor Analysis Factor Loadings, Communalities (h2) and Cronbach’s a if Item Deleted for the Five-factor Model of the ED-CBQ in the Full Sample and ED-symptomatic subgroup.

**Additional file 3: Table B.** Confirmatory Factor Analysis Factor Loadings, Communalities (h2) and Cronbach’s a if Item Deleted for the ED-CBQ-SF and ED-CBQ-R.

**Additional file 4: Table C.** Descriptive Statistics for the ED-CBQ and ED-CBQ-R in the Full Sample, Likely-ED and non-ED Subgroups.

## Data Availability

The datasets used and/or analysed during the current study available from the corresponding author on reasonable request.

## Abbreviations

AN: Anorexia Nervosa
ANOVA: Analysis of Variance
APA: American Psychiatric Association
AUC: Area under the curve
BED: Binge Eating Disorder
BMI: Body Mass Index
BN: Bulimia Nervosa
BSQ: Body Shape Questionnaire
CBT: Cognitive Behavioural Therapy
CFA: Confirmatory Factor Analysis
CFI: Comparative Fit Index
CI: Confidence Intervals
DASS-21: Depression Anxiety Stress Scale 21
DEBQ: Dutch Eating Behaviour Questionnaire
EBQ-18: Eating Beliefs Questionnaire 18
ED: Eating Disorder
EDBQ: Eating Disorder Beliefs Questionnaire
ED-CBQ: Eating Disorders Core Beliefs Questionnaire
EDE: Eating Disorders Examination
EDE-Q: Eating Disorder Examination Questionnaire
EFA: Exploratory Factor Analysis
NFI: Normed fit index
NPV: Negative predictive value
OSFED: Other Specified Feeding and Eating Disorders
PPV: Positive predictive value
RMSEA: Root Mean Square Error of Approximation
RFI: Relative fit index
ROC: Receiver operating characteristic curve
SD: Standard Deviation
SPSS: Statistical Package for Social Sciences
TLI: Tucker-Lewis Index
YSQ: Young Schema Questionnaire
χ^2^/df: Chi-square/degrees of freedom

## Footnotes

1 A five-factor 18-item model was also tested, with the initial intention of retaining the authors original five-factor structure (see Additional file 3: Table B). Despite the five-factor 18-item model displaying superior model fit compared to the original 40-item measure, the internal consistency of this model was substantially increased when removing all items belonging to High Standards for Self. Upon examination of both the reliability outcomes and the model fit statistics, in conjunction with examining inter-subscale correlations and group differences, we then tested the alternate four-factor structure model by removing High Standards for Self entirely from the revision process.

2 Supplementary analyses were conducted to compare how individuals with a likely ED diagnosis would compare to a non-ED group. Additional file 4: Table C displays the mean scores and t-test results showing group differences between a non-ED subgroup to a likely-ED subgroup (using the more stringent EDE-Q global score cut-off of 4.0) for interested readers.
