# Supplementary material for: Assessing negative core beliefs in eating disorders: revision of the Eating Disorder Core Beliefs Questionnaire
Source: J Eat Disord. 2022 Feb 10;10:18. doi: 10.1186/s40337-022-00542-9 (PMC8830168; doi:10.1186/s40337-022-00542-9)
Supplement: Supplementary file 1 — Additional file 1: File 1. Eating Disorder Core Beliefs Questionnaire - Revised (ED-CBQ-R). [file 40337_2022_542_MOESM1_ESM.docx]

**Eating Disorder Core Beliefs Questionnaire - Revised (ED-CBQ-R)**

Listed below are a number of different words. People sometimes think these words describe how they feel about themselves as a person. Please read each word carefully and decide how much you feel each word describes how you feel about yourself. Base your answer on what you emotionally believe or feel to be true, not on what you rationally believe to be true. Choose the rating which best describes what you usually believe/feel or what you believe/feel most of the time, rather than how you feel right now. If you are unsure of the meaning of a word you may skip it. Work as quickly as you can. Don't spend too long on each word—your first impression is most important. Place a cross or tick in the box that best describes your response.

|  | | **Feels**  **very much untrue** | **Feels moderately untrue** | **Feels slightly untrue** | **Feels neither true nor untrue** | **Feels slightly true** | **Feels moderately true** | **Feels**  **very much**  **true** |
| --- | --- | --- | --- | --- | --- | --- | --- | --- |
| **1** | **Abandoned** |  |  |  |  |  |  |  |
| **2** | **Betrayed** |  |  |  |  |  |  |  |
| **3** | **Complaining** |  |  |  |  |  |  |  |
| **4** | **Deprived** |  |  |  |  |  |  |  |
| **5** | **Inhibited** |  |  |  |  |  |  |  |
| **6** | **Meek** |  |  |  |  |  |  |  |
| **7** | **Needy** |  |  |  |  |  |  |  |
| **8** | **Possessive** |  |  |  |  |  |  |  |
| **9** | **Putrid** |  |  |  |  |  |  |  |
| **10** | **Repugnant** |  |  |  |  |  |  |  |
| **11** | **Repulsive** |  |  |  |  |  |  |  |
| **12** | **Selfish** |  |  |  |  |  |  |  |
| **13** | **Submissive** |  |  |  |  |  |  |  |
| **14** | **Unassertive** |  |  |  |  |  |  |  |
| **15** | **Vile** |  |  |  |  |  |  |  |

**Scoring:**

Subscale 1: Self Loathing = Items (9 + 10 + 11 + 15) / 4

Subscale 2: Unassertive/Inhibited = Items (5 + 6 + 13 + 14) / 4

Subscale 3: Demanding = Items (3 + 7 + 8 + 12) / 4

Subscale 4: Abandoned = Items (1 + 2 + 4) / 3

^1^To cite this measure please refer to the development paper: Hatoum, Burton & Abbott (2022)

^2^This measure is based off the original 40-item ED-CBQ by Fairchild & Cooper (2010). Fairchild, H., & Cooper, M. (2010). A multidimensional measure of core beliefs relevant to eating disorders: Preliminary development and validation. *Eating Behaviors*, *11*(4), 239–246. https://doi.org/10.1016/j.eatbeh.2010.05.004
