# Supplementary material for: Assessing negative core beliefs in eating disorders: revision of the Eating Disorder Core Beliefs Questionnaire
Source: J Eat Disord. 2022 Feb 10;10:18. doi: 10.1186/s40337-022-00542-9 (PMC8830168; doi:10.1186/s40337-022-00542-9)
Supplement: Supplementary file 2 — Additional file 2: Table A. Confirmatory Factor Analysis Factor Loadings, Communalities (h2) and Cronbach’s a if Item Deleted for the Five-factor Model of the ED-CBQ in the Full Sample and ED-symptomatic subgroup. [file 40337_2022_542_MOESM2_ESM.docx]

**Supplementary Table A**

*Confirmatory Factor Analysis Factor Loadings, Communalities (h^2^) and Cronbach’s α if Item Deleted for the Five-factor Model of the ED-CBQ in the Full Sample and ED-symptomatic subgroup*

| **Item** | **Full Sample (*n* = 763)** | | | **ED-symptomatic Subgroup (*n* = 384)** | | |
| --- | --- | --- | --- | --- | --- | --- |
|  | **Factor Loadings** | ***h^2^*** | **α if Item Deleted** | **Factor Loadings** | ***h^2^*** | **α if Item Deleted** |
| **Self-Loathing** |  |  |  |  |  |  |
| Disgraceful | .73 | .60 | .92 | .72 | .57 | .93 |
| Evil | .69 | .54 | .92 | .72 | .61 | .93 |
| Filthy | .72 | .52 | .92 | .71 | .53 | .93 |
| Inhuman | .67 | .49 | .93 | .70 | .53 | .93 |
| Nasty | .68 | .55 | .93 | .68 | .56 | .93 |
| Poisonous | .72 | .53 | .92 | .74 | .58 | .93 |
| Putrid | .85 | .77 | .92 | .86 | .82 | .92 |
| Repugnant | .85 | .71 | .92 | .84 | .75 | .92 |
| Repulsive | .82 | .68 | .92 | .83 | .73 | .92 |
| Vile | .85 | .75 | .92 | .86 | .77 | .92 |
| **Unassertive** |  |  |  |  |  |  |
| Inhibited | .55 | .37 | .79 | .55 | .39 | .76 |
| Meek | .61 | .41 | .778 | .56 | .44 | .75 |
| Reserved | .47 | .31 | .80 | .38 | .30 | .78 |
| Submissive | .64 | .41 | .77 | .57 | .41 | .75 |
| Unassertive | .77 | .56 | .76 | .72 | .58 | .73 |
| Undemonstrative | .80 | .60 | .76 | .79 | .61 | .73 |
| Unemotional | .37 | .40 | .81 | .38 | .51 | .78 |
| Unreflective | .47 | .36 | .79 | .50 | .57 | .76 |
| **Demanding** |  |  |  |  |  |  |
| Complaining | .66 | .49 | .83 | .64 | .50 | .84 |
| Demanding | .60 | .47 | .83 | .57 | .57 | .84 |
| Ill-tempered | .60 | .37 | .84 | .60 | .38 | .84 |
| Immature | .46 | .27 | .85 | .53 | .40 | .85 |
| Manipulative | 51 | .35 | .84 | .57 | .44 | .84 |
| Needy | .65 | .47 | .83 | .61 | .49 | .84 |
| Possessive | .62 | .38 | .84 | .63 | .44 | .84 |
| Selfish | .69 | .49 | .83 | .71 | .54 | .83 |
| Superficial | .64 | .41 | .83 | .62 | .44 | .84 |
| Suspicious | .60 | .36 | .84 | .60 | .37 | .84 |
| **Abandoned** |  |  |  |  |  |  |
| Abandoned | .80 | .44 | .66 | .80 | .73 | .64 |
| Betrayed | .73 | .35 | .70 | .74 | .54 | .67 |
| Deprived | .67 | .41 | .73 | .65 | .41 | .72 |
| Misunderstood | .56 | .38 | .78 | .52 | .36 | .78 |
| **High Standards for Self** |  |  |  |  |  |  |
| Conscientious | .36 | .26 | .75 | .38 | .26 | .73 |
| Focused | .83 | .60 | .73 | .84 | .66 | .71 |
| Goal-oriented | .82 | .61 | .73 | .84 | .68 | .71 |
| Meticulous | .33 | .37 | .73 | .27 | .70 | .72 |
| Painstaking | -.03 | .42 | .79 | -.07 | .46 | .78 |
| Perfectionistic | .46 | .43 | .72 | .44 | .44 | .70 |
| Persistent | .55 | .44 | .72 | .54 | .49 | .70 |
| Self-disciplined | .61 | .42 | .74 | .57 | .42 | .73 |

*Note.* Cronbach’s α if item deleted refers to the item being removed from its subscale, not from the overall scale. ED = Eating Disorder.
