# Supplementary material for: Assessing negative core beliefs in eating disorders: revision of the Eating Disorder Core Beliefs Questionnaire
Source: J Eat Disord. 2022 Feb 10;10:18. doi: 10.1186/s40337-022-00542-9 (PMC8830168; doi:10.1186/s40337-022-00542-9)
Supplement: Supplementary file 3 — Additional file 3: Table B. Confirmatory Factor Analysis Factor Loadings, Communalities (h2) and Cronbach’s a if Item Deleted for the ED-CBQ-SF and ED-CBQ-R. [file 40337_2022_542_MOESM3_ESM.docx]

**Supplementary Table B**

*Confirmatory Factor Analysis Factor Loadings, Communalities (h^2^) and Cronbach’s α if Item Deleted for the ED-CBQ-SF and ED-CBQ-R*

| **Item** | **ED-CBQ-SF**  **Full Sample**  **(*n* = 763)** | | | **ED-CBQ-SF**  **ED-symptomatic Subgroup**  **(*n* = 384)** | | | **ED-CBQ-R**  **Full Sample**  **(*n* = 763)** | | **ED-CBQ-R**  **ED-symptomatic Subgroup**  **(*n* = 384)** | |
| --- | --- | --- | --- | --- | --- | --- | --- | --- | --- | --- |
|  | **Factor Loadings** | ***h^2^*** | **α if Item Deleted** | **Factor Loadings** | ***h^2^*** | **α if Item Deleted** | **Factor Loadings** | ***h^2^*** | **Factor Loadings** | ***h^2^*** |
| **Self-Loathing** |  |  |  |  |  |  |  |  |  |  |
| Putrid | .90 | .84 | .90 | .84 | .89 | .91 | .90 | .84 | .92 | .86 |
| Repugnant | .89 | .79 | .89 | .79 | .89 | .88 | .89 | .79 | .88 | .77 |
| Repulsive | .87 | .75 | .87 | .75 | .90 | .87 | .87 | .74 | .87 | .76 |
| Vile | .82 | .67 | .82 | .67 | .92 | .82 | .82 | .67 | .82 | .68 |
| **Unassertive** |  |  |  |  |  |  |  |  |  |  |
| Inhibited | .59 | .35 | .59 | .35 | .73 | .62 | .59 | .36 | .62 | .39 |
| Meek | .65 | .48 | .65 | .48 | .67 | .60 | .65 | .49 | .59 | .45 |
| Submissive | .69 | .50 | .69 | .50 | .67 | .60 | .69 | .49 | .60 | .42 |
| Unassertive | .68 | .47 | .68 | .47 | .67 | .63 | .68 | .46 | .63 | .41 |
| **Demanding** |  |  |  |  |  |  |  |  |  |  |
| Complaining | .66 | .44 | .66 | .44 | .70 | .63 | .66 | .44 | .63 | .39 |
| Needy | .71 | .54 | .71 | .54 | .66 | .68 | .71 | .54 | .68 | .56 |
| Possessive | .63 | .40 | .63 | .40 | .71 | .64 | .63 | .39 | .64 | .41 |
| Selfish | .62 | .39 | .62 | .39 | .70 | .65 | .62 | .40 | .65 | .42 |
| **Abandoned** |  |  |  |  |  |  |  |  |  |  |
| Abandoned | .83 | .72 | .83 | .72 | .62 | .83 | .83 | .73 | .83 | .72 |
| Betrayed | .75 | .59 | .75 | .59 | .69 | .75 | .76 | .57 | .75 | .56 |
| Deprived | .65 | .44 | .65 | .44 | .80 | .64 | .65 | .44 | .64 | .41 |
| **High Standards for Self** |  |  |  |  |  |  |  |  |  |  |
| Focused | .87 | .74 | .87 | .74 | .65 | .87 | - | - | - | - |
| Goal-oriented | .83 | .73 | .83 | .73 | .67 | .85 | - | - | - | - |
| Self-disciplined | .58 | .36 | .58 | .36 | .84 | .53 | - | - | - | - |

*Note.* Cronbach’s α if item deleted refers to the item being removed from its subscale, not from the overall scales. ED = Eating Disorder; ED-CBQ = Eating Disorder Core Beliefs Questionnaire; ED-CBQ-SF = Eating Disorder Core Beliefs Questionnaire Short Form; ED-CBQ-R = Eating Disorder Core Beliefs Questionnaire Revised.
