# Supplementary material for: Assessing negative core beliefs in eating disorders: revision of the Eating Disorder Core Beliefs Questionnaire
Source: J Eat Disord. 2022 Feb 10;10:18. doi: 10.1186/s40337-022-00542-9 (PMC8830168; doi:10.1186/s40337-022-00542-9)
Supplement: Supplementary file 4 — Additional file 4: Table C. Descriptive Statistics for the ED-CBQ and ED-CBQ-R in the Full Sample, Likely-ED and non-ED Subgroups. [file 40337_2022_542_MOESM4_ESM.docx]

**Supplementary Table C**

*Descriptive Statistics for the ED-CBQ and ED-CBQ-R in the Full Sample, Likely-ED and non-ED Subgroups*

|  | **Full Sample**  **(*N* = 763)** | **Likely-ED subgroup**  **(*n* = 157)** | **Non-ED subgroup**  **(*n* = 606)** | ***t*** | ***p*** | ***d*** |
| --- | --- | --- | --- | --- | --- | --- |
| **Total ED-CBQ Mean (SD)** | 3.04 (0.82) | 3.46 (0.88) | 2.93 (0.76) | 6.98 | <.001 | .68 |
| Self-Loathing Mean (SD) | 1.85 (1.08) | 2.54 (1.42) | 1.67 (0.90) | 7.30 | <.001 | .85 |
| Unassertive Mean (SD) | 3.04 (1.13) | 3.35 (1.12) | 2.96 (1.12) | 3.91 | <.001 | .35 |
| Demanding Mean (SD) | 3.23 (1.19) | 3.75 (1.22) | 3.10 (1.14) | 6.11 | <.001 | .57 |
| Abandoned Mean (SD) | 2.82 (1.37) | 3.56 (1.44) | 2.63 (1.28) | 7.39 | <.001 | .71 |
| High Standards for Self Mean (SD) | 4.39 (1.05) | 4.32 (1.04) | 4.41 (1.05) | 0.91 | .362 | .08 |
| **Total ED-CBQ-R Mean (SD)** | 2.71 (1.05) | 3.34 (1.12) | 2.54 (0.97) | 8.13 | <.001 | .79 |
| Self-Loathing Mean (SD) | 1.75 (1.19) | 2.51 (1.64) | 1.55 (0.95) | 7.06 | <.001 | .86 |
| Unassertive Mean (SD) | 3.12 (1.37) | 3.50 (1.27) | 3.02 (1.37) | 4.19 | <.001 | .36 |
| Demanding Mean (SD) | 3.45 (1.40) | 4.04 (1.39) | 3.30 (1.36) | 6.01 | <.001 | .54 |
| Abandoned Mean (SD) | 2.45 (1.46) | 3.27 (1.61) | 2.23 (1.34) | 7.44 | <.001 | .74 |

*Note.* *t*-values indicates the difference in scores between the Likely-ED and non-ED subgroups using Welch’s *t-*test where equal variances were not assumed. The Likely-ED group was created using an EDE-Q global cut-off score of 4. ED = Eating Disorder; ED-CBQ = Eating Disorder Core Beliefs Questionnaire; ED-CBQ-R = Eating Disorder Core Beliefs Questionnaire Revised; SD = Standard Deviation. Scores ranged from 1 to 7, with higher scores indicating higher endorsement of each subscale.
